# Role and mechanisms of SGLT-2 inhibitors in the treatment of diabetic kidney disease

**DOI:** 10.3389/fimmu.2023.1213473

**Published:** 2023-09-21

**Authors:** Zhi-Cheng Dai, Jin-Xia Chen, Rong Zou, Xuan-Bing Liang, Ji-Xin Tang, Cui-Wei Yao

**Affiliations:** Guangdong Provincial Key Laboratory of Autophagy and Major Chronic Non-communicable Diseases, Key Laboratory of Prevention and Management of Chronic Kidney Diseases of Zhanjiang City, Institute of Nephrology, Affiliated Hospital of Guangdong Medical University, Zhanjiang, Guangdong, China

**Keywords:** SGLT-2 inhibitor, diabetic kidney disease, renal protection mechanism, efficacy, safety

## Abstract

Diabetic kidney disease (DKD) is a chronic inflammatory condition that affects approximately 20-40% of individuals with diabetes. Sodium-glucose co-transporter 2 (SGLT-2) inhibitors, emerging as novel hypoglycemic agents, have demonstrated significant cardiorenal protective effects in patients with DKD. Initially, it was believed that the efficacy of SGLT-2 inhibitors declined as the estimated glomerular filtration rate (eGFR) decreased, which led to their preferential use in DKD patients at G1-G3 stages. However, recent findings from the DAPA-CKD and EMPA-KIDNEY studies have revealed equally beneficial cardiorenal effects of SGLT-2 inhibitors in individuals at stage G4 DKD, although the underlying mechanism behind this phenomenon remains unclear. In this comprehensive analysis, we provide a systematic review of the mechanisms and functioning of SGLT-2 inhibitors, potential renal protection mechanisms, and the therapeutic efficacy and safety of SGLT-2 inhibitors in kidney diseases, with a particular focus on stage G4 DKD. Gaining a deeper understanding of the renal protective effect of SGLT-2 inhibitors and their underlying mechanisms is highly significance for the successful utilization of these inhibitors in the treatment of diverse kidney disorders.

## 1 Introduction

Diabetes, a prevalent illness worldwide, poses a significant threat to human health. As of 2019, approximately 463 million individuals were affected by diabetes, and this number is projected to reach 700 million by 2045 (1). Diabetic kidney disease (DKD), a common microvascular complication associated with diabetes, affects around 20-40% of diabetic patients and can progress to end-stage renal disease (ESRD) in some cases (2–4). While controlling blood glucose levels is crucial in slowing DKD progression, it becomes challenging to lower glycated hemoglobin in patients with advanced DKD, leading to difficulties in halting disease advancement due to patient or pharmaceutical factors (5). Consequently, the pursuit of effective therapeutic approaches that can safeguard kidney function and delay the onset of DKD has emerged as a prominent research area in DKD studies.

Currently, renin-angiotensin system inhibitors are recommended as the primary drugs per the guidelines for treating DKD. However, they are not entirely adequate for DKD treatment due to their potential increased risk of ESRD and acute renal failure (6, 7). The effectiveness and safety of SGLT-2 inhibitors, a novel hypoglycemic agent, have been validated for stage G1-G3 DKD therapy, resulting in the 2020 KDIGO guidelines recommending their use for DKD patients with an estimated glomerular filtration rate (eGFR) ≥ 30 mL/min/1.73 m^2^ (8). Further research is still necessary to ascertain their efficacy and safety for individuals with stage G4-G5 DKD.

Recent findings from the DAPA-CKD and EMPA-KIDNEY trials have revealed that SGLT-2 inhibitors offer significant benefits in improving cardiovascular and renal function, as well as delaying the progression of renal disease in stage G4 DKD patients, irrespective of their diabetes status (9, 10). As a result, the 2022 KDIGO guidelines have also recommended a revision of the eGFR threshold to 20 mL/min/1.73 m^2^ for DKD patients (11). Therefore, this paper aims to comprehensively review the research progress surrounding the mechanisms of action of SGLT-2 inhibitors, their potential renal protective effects, and their efficacy and safety in patients with stage G4 DKD.

## 2 The function of SGLT-2 inhibitors

### 2.1 SGLT-2 inhibitors and mechanism of action

**Figure 1:**
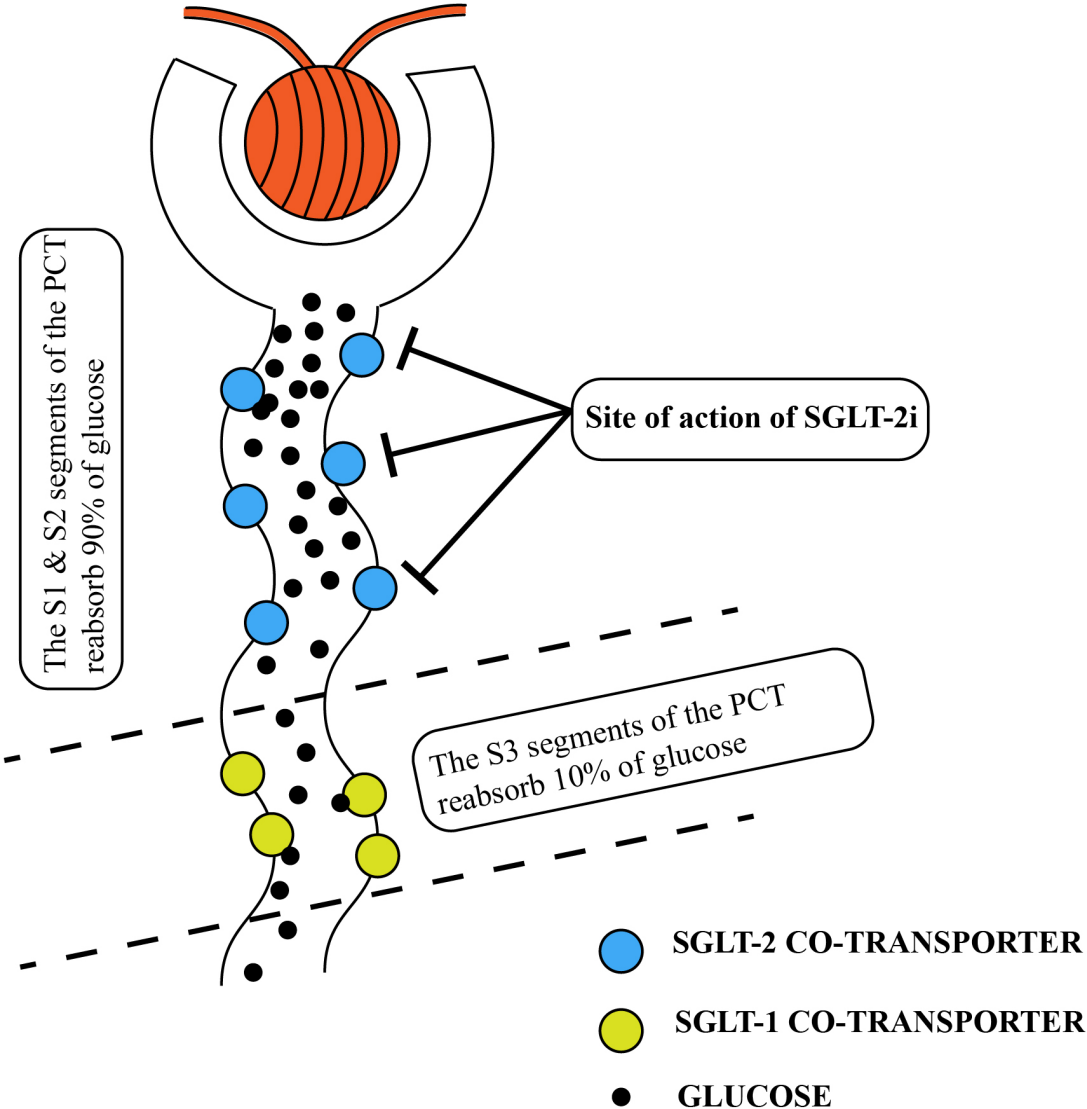
Normal renal tubular resorption of glucose. The location of the SGLT2-inhibitor’s action is also shown in the diagram. PCT, proximal convoluted tubules; SGLT, sodium-glucose co-transporter.

SGLT-2 inhibitors are a relatively new class of antidiabetic medications that have gained attention in recent years. The use of non-selective SGLT inhibitors, extracted from apple tree root bark glycosides, was first reported in the 1830s and since then, SGLT-2 inhibitors have become a popular focus of research due to their unique mechanism of action, which does not rely on insulin (12). SGLT-1 and SGLT-2 are crucial molecules involved in glucose reabsorption in the kidney and are primarily located in the renal tubular epithelium. In normal physiological conditions, glucose filtered from the glomerulus enters the tubules and is reabsorbed by SGLT-1 and SGLT-2. Among these transporters, SGLT-2 is predominant in the S1 and S2 segments of the renal proximal tubule and functions as a high-capacity glucose transporter, responsible for approximately 90% of glucose reabsorption in the renal tubules. On the other hand, SGLT-1 functions as a low-capacity glucose transporter and is found not only in the kidneys but also in the gastrointestinal tract, where it plays a role in reabsorbing a smaller amount of glucose in the renal tubules (13) (**Figure 1**).

SGLT-2 inhibitors are a class of hypoglycemic agents that have gained popularity in recent years. They work by competing with the SGLT-2 protein for glucose binding in the renal tubules. This competition prevents SGLT-2 from binding to glucose, leading to reduced glucose reabsorption by the renal tubules (**Figure 1**). As a result, there is an increased excretion of glucose, sodium, and water in the urine. This leads to lower blood glucose and reduced volume load (14). Importantly, the mechanism of action of SGLT-2 inhibitors is independent of insulin regulation. It does not rely on the regulation of insulin secretion by pancreatic β-cells or insulin resistance in the body. Currently, there are several selective SGLT-2 inhibitors available on the market, such as empagliflozin, dapagliflozin, and luseogliflozin. These medications specifically inhibit SGLT-2. However, there are also SGLT-1 and SGLT-2 inhibitors, such as sotagliflozin and canagliflozin, which can inhibit both transporters (15). Overall, the discovery and development of SGLT-2 inhibitors have provided a novel approach for managing blood glucose levels in individuals with diabetes.

### 2.2 Hypoglycemic effects

The hypoglycemic effect of SGLT-2 inhibitors relies on renal tubular reabsorption, and thus the amount of glucose excretion in the urine is directly related to the glomerular filtration rate (16, 17). In individuals with advanced DKD, specifically those with stage G4-G5 DKD, the options for hypoglycemic medications are limited, and dosage is often restricted. Therefore, it is crucial to assess the effectiveness of treating patients with relatively advanced DKD using SGLT-2 inhibitors. Several clinical studies have focused on the glycemic efficacy of SGLT-2 inhibitors in individuals with type 2 diabetes and stage 3 chronic kidney disease (CKD). Their findings indicate that the glucose-lowering effect is attenuated compared to patients with normal renal function (18–20). In a study involving dapagliflozin treatment for patients with type 2 diabetes and stage 3b-4 CKD, glycated hemoglobin levels did not decrease over the 102-week treatment period (21). Similarly, another study focusing on DKD patients treated with luseogliflozin found that the increase in urinary glucose excretion was lower in those with stage G4 DKD compared to patients with stage G1-G3 DKD (22). These findings suggest that the hypoglycemic efficacy of SGLT-2 inhibitors is reduced in patients with stage G4 DKD when compared to those with stage G1-G3 DKD.

### 2.3 Nephroprotective effects

#### 2.3.1 Reduction of glomerular hyperfiltration

On one hand, SGLT-2 receptors are primarily found in the proximal tubule and are responsible for the reabsorption of glucose and sodium ions. Consequently, the decreased sodium concentration sensed at the macula densa location triggers a tubuloglomerular feedback (TGF) mechanism, which stimulates contraction of the efferent arteries and dilation of the afferent arteries. This results in increased blood flow into the glomerulus, raising the glomerular pressure and leading to glomerular hyperfiltration and subsequent damage (23). In contrast, SGLT-2 inhibitors can reduce Na^+^ reabsorption in the renal tubules by competitively binding glucose. This restoration of the TGF mechanism promotes relaxation of the small arterial outflow, reducing intraglomerular pressure and alleviating glomerular hyperfiltration. These effects are crucial in preserving renal function and slowing the progression of nephropathy (24).

However, another important player in Na^+^ reabsorption in the proximal tubule is the Na^+^-H^+^ exchanger 3 (NHE3), responsible for about 70% of Na^+^ reabsorption through direct or indirect mechanisms (25). Activation of the TGF system leads to increased intraglomerular pressure and subsequent glomerular hyperfiltration. Recent research has demonstrated that NHE3 works synergistically with SGLT-2 in Na^+^ reabsorption, with SGLT-2 tightly regulating NHE3 activity. Therefore, NHE3 is sensitive to the modulation by SGLT-2 inhibitors (26, 27). A study conducted on diabetic mice revealed that SGLT-2 inhibitors have the ability to inhibit NHE3 function by promoting NHE3 phosphorylation. This phosphorylation enhances Na^+^ excretion, effectively reducing sodium levels within the body (28).This finding demonstrates the potential of SGLT-2 inhibitors as therapeutic agents in regulating NHE3 activity and mitigating glomerular hyperfiltration.

Hence, the underlying mechanism behind the natriuretic effect of SGLT-2 inhibitors is to combat sodium-water retention and repair TGF system, thereby alleviating the condition of glomerular hyperfiltration for renal protection. This effect could be attributed to the competitive inhibition of SGLT receptors and the subsequent inhibition of NHE3. Notably, NHE3 seems to play a pivotal role in the overall natriuretic effect of SGLT-2 inhibitors.

#### 2.3.2 Regulation of the renin-angiotensin-aldosterone system (RAAS)

The activation of RAAS, particularly the increased secretion of angiotensin II (Ang II) and aldosterone, has been established as a crucial factor in the progression of DKD (29–31). In response to elevated blood glucose levels, SGLT-2 in the proximal renal tubule enhances Na^+^ and glucose reabsorption. This leads to reduced sensing of Na^+^ by the macula densa, resulting in decreased adenosine production, which further activates the RAAS and causes constriction of the small afferent arteries. Moreover, the enhanced production of Ang II, stimulated by RAAS activation, also triggers the release of aldosterone. The resulting oxidative stress, inflammation, and renal fibrosis contribute to the accelerated progression of DKD (32). With the usage of SGLT2 inhibitors, the macula densa becomes more sensitive to changes in Na^+^ concentration. This results in increased adenosine production and suppression of RAAS activation. Consequently, there is vasoconstriction in the afferent arteries, while the inhibition of RAAS also reduces the release of aldosterone release. These combined effects help to alleviate oxidative stress, inflammation, and fibrosis in the kidney, thereby mitigating the progression of DKD (33).

In the past few decades, RAAS inhibitors have been widely used as the primary approach to treat DKD (34). However, the distinction between SGLT-2 inhibitors and RAAS inhibitors lies in their differential effects on the small afferent and efferent glomerular arteries. RAAS inhibitors dilate both the afferent and efferent arterioles, but the efferent arterioles are dilated to a greater extent, resulting in continued glomerular ultrafiltration and subsequent damage to kidney units and podocytes (35). Consequently, RAAS inhibitors alone may not effectively halt the progression of early-stage DKD or provide optimal treatment for individuals with advanced DKD (29, 36). However, SGLT-2 inhibition provides a greater degree of renal protection compared to RAAS inhibition as it not only relieves glomerular hyperfiltration but also reduces intra-glomerular pressure by dilating small outgoing glomerular arteries and constricting small incoming glomerular arteries. This unique mechanism of action suggests that SGLT-2 inhibitors may offer enhanced efficacy in delaying the progression of DKD.

## 3 Potential renal protection mechanisms

**Figure 2:**
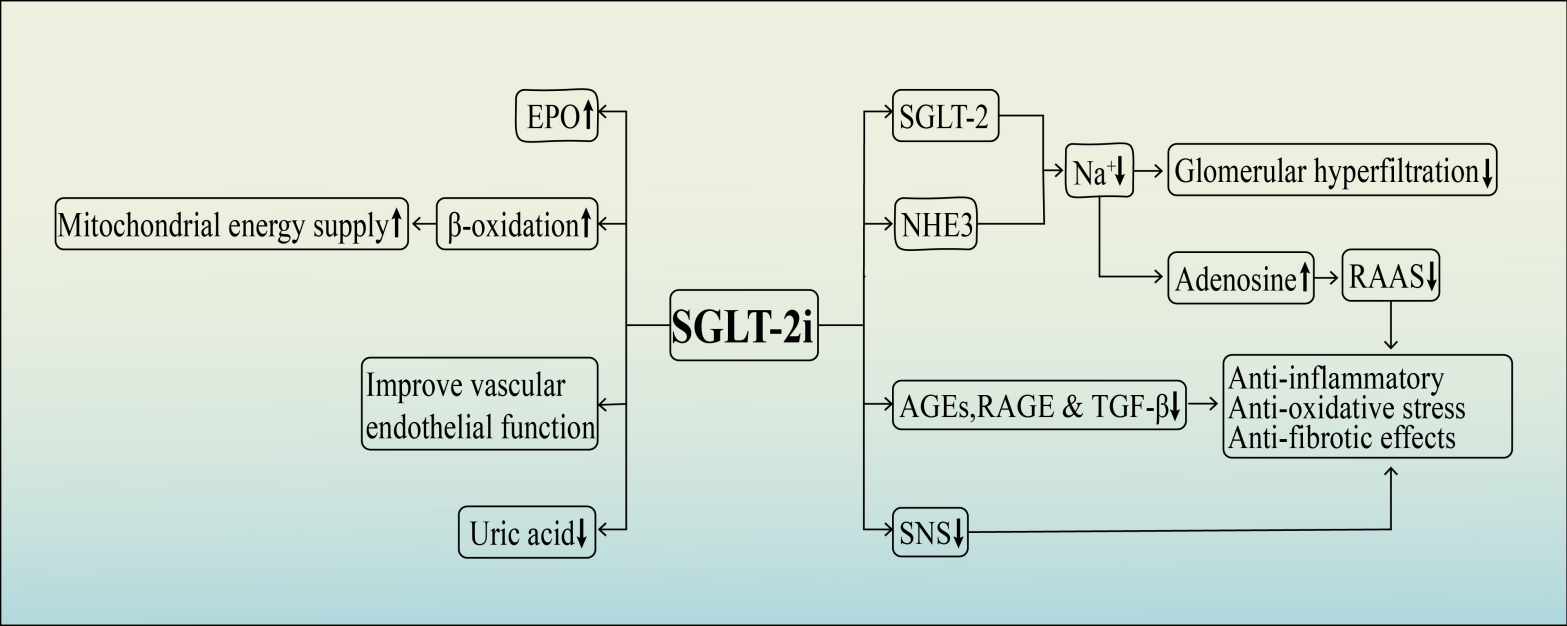
The diagram shows the renal protection mechanism of SGLT-2 inhibitors.

Although the exact mechanisms by which SGLT-2 inhibitors protect the kidneys are not fully understood, recent research has shown that these medications have numerous potential renoprotective effects in addition to their traditional hypoglycemic effects. These effects include reducingglomerular hyperfiltration and volume load, inhibiting inflammation and fibrosis, improving oxidative stress, enhancing erythropoietin (EPO) production, enhancing mitochondrial energy supply, inhibiting the sympathetic nervous system, protecting vascular endothelial cells, and reducing blood uric acid levels, among others (37–42) (**Figure 2**).

### 3.1 Anti-inflammatory, anti-oxidative stress and anti-fibrotic effects

Mild systemic inflammation is commonly observed in patients with DKD. Prolonged hyperglycemia leads to the production of advanced glycosylation end products (AGEs) through the glycosylation of non-enzymatic proteins. These AGEs promote the formation of receptors for advanced glycosylation end products (RAGE), resulting in oxidative stress and increased levels of reactive oxygen species. This oxidative stress contributes to the progression of kidney fibrosis and further decline in renal function, marking a crucial phase in the development of DKD (43, 44). One of the key pro-fibrotic factors involved in this process is transforming growth factor 1 (TGF-1). TGF-1, along with other pro-fibrotic factors, intensifies inflammation and exerts strong pro-fibrotic effects. It promotes the proliferation of mesangial cells and the deposition of extra-mesenchymal matrix, leading to glomerulosclerosis and interstitial fibrosis (45).

In a study by Ojima et al., diabetic rats were used to investigate the effects of empagliflozin on renal tissues. After 4 weeks of empagliflozin treatment, there was a significant reduction in the expression of AGEs, RAGE, and other proteins in renal tissues. This resulted in the inhibition of the AGE-RAGE oxidative stress axis, demonstrating empagliflozin’s potential as an anti-inflammatory, anti-fibrotic, and tubular protective agent (46). Further experiments revealed that empagliflozin effectively mitigated oxidative stress and attenuated damage to the renal tissues. It is believed that this protective effect may be attributed to empagliflozin’s ability to downregulate the TGF-β-Smad pathway and upregulate the Nrf2/ARE pathway (47). Consequently, SGLT-2 inhibitors such as empagliflozin have the potential to safeguard kidney health by suppressing the expression of AGEs, RAGE, and TGF-β1, thereby exerting anti-inflammatory, anti-oxidative stress, and anti-fibrotic effects.

### 3.2 Promotion of EPO production

Renal anemia is a prevalent complication in advanced DKD, and its management has been shown to slow down the progression of renal failure (48, 49). In diabetic patients, increased glucose reabsorption through SGLT-2 by proximal tubular epithelial cells results in excessive consumption of adenosine triphosphate (ATP) produced by the Na^+^/K^+^ pump. This leads to heightened oxygen consumption by tubular epithelial cells and subsequently reduced oxygen partial pressure in the kidney’s cortical tissue. The hypoxic microenvironment of renal tubular epithelial cells triggers the transformation of EPO-producing fibroblasts into myofibroblasts, consequently reducing EPO production (50, 51). By diminishing glucose reabsorption and lowering the demand on the Na^+^/K^+^ pump ATP, SGLT-2 inhibitors alleviate metabolic stress on proximal tubules, thereby improving the microenvironment within renal tubules.

This leads to the transformation of myofibroblasts to fibroblasts, which partially increases EPO production and contributes to the correction of anemia (52). The EMPA-HEART CardioLink-6 trial demonstrated that empagliflozin-induced early elevation of EPO and subsequent increments in hematocrit correlated with reductions in ferritin and hemoglobin levels (53). An analysis of data from the EMPA-REG outcome trial suggests that approximately 50% of the mortality benefits associated with increased hematocrit in SGLT-2 inhibitor-treated patients may be attributed to enhanced erythropoietin production. This gain might be due to the protective effects of EPO as a circulating pleiotropic cytokine, including promoting angiogenesis, improvingmitochondrial function, and suppressing inflammation. Moreover, the elevated hematocrit resulting from EPO also directly enhances the oxygen-carrying capacity of systemic tissues (54). Hence, by stimulating the production of EPO,SGLT-2 inhibitors have the potential to safeguard renal function and slow down the advancement of renal failure.

### 3.3 Improved mitochondrial energy supply

The renal tubule’s epithelial cells contain abundant mitochondria, which play a crucial role in supplying energy for the reabsorption of metabolic substances like glucose and Na^+^ in the renal tubules. ATP, produced by mitochondria, is essential for glucose uptake by tubular epithelial cells. Glucose and fatty acids are the primary sources of substrates for ATP production, with β-oxidation of fatty acids generating significantly more ATP than aerobic fermentation of glucose (55, 56). In a hyperglycemic environment, there is an increase in glycolysis, tricarboxylic acid cycle, and β-oxidation, along with an elevation in oxygen free radical production, leading to tubulointerstitial fibrosis and hastening the progression of renal failure (57, 58). Research indicatesthat impaired fatty acid oxidation occurs in parallel with the advancement of DKD (59, 60). Hence, enhancing mitochondrial fatty acid oxidation to ensure a more efficient energy supply could potentially delay the progression of DKD.

SGLT-2 inhibitors have been found to increase urinary glucose excretion, leading to a decrease in glucose levels in the body. This causes a shift in energy utilization, where fatty acid β-oxidation is employed as the main source of energy. This shift in energy supply is primarily responsible for the weight loss observed with SGLT-2 inhibitors (61). Furthermore, increased β-oxidation results in the production of excess acetyl coenzyme A, which generates ketone bodies (β-hydroxybutyrate, acetoacetate, and acetone). These ketone bodies serve as a fuel source for ATP production in the mitochondria, thus improving mitochondrial energy supply (62, 63). Moreover, SGLT-2 inhibitors can enhance mitochondrial energy supply through various mechanisms. They promote EPO production, enhance renal tissue oxygenation, and facilitate the conversion of synthetic ATP fuel from glucose to ketone bodies (64). Numerous clinical studies have demonstrated that the application of SGLT-2 inhibitors promotes the synthesis of ketone body and decreases the insulin-to-glucagon ratio (65–67). Hence, it is believed that SGLT-2 inhibitors have the potential to enhance kidney function by augmenting ketone body production through β-oxidation, thereby improving the mitochondrial energy supply to the renal tubular epithelial cells.

### 3.4 Inhibition of the sympathetic nervous system (SNS)

Persistent activation of the SNS has been strongly linked to the onset of type 2 diabetes (68). Moreover, its excessive activation is associated with a negative prognosis in patients suffering from advanced DKD (69). SNS hyperactivity has been correlated with glomerulosclerosis, protein loss, and microalbuminuria. Additionally, it promotes the development of renal fibrosis by triggering pro-inflammatory and pro-fibrotic markers such as tumor necrosis factor-β, it also hastens the development of renal fibrosis. This, in turn, leads to a decline in glomerular filtration rate and further exacerbates SNS activation, creating a harmful cycle that accelerates the progression of DKD (70, 71). Sano has proposed that the kidneys play a central role in sympathetic overactivation and has suggested that SGLT-2 inhibitors could potentially provide cardiovascular and renal benefits by reducing afferent renal nerve activity and inhibiting reflex mechanisms within the central nervous system that activate the systemic sympathetic nerves (72). Herat et al. proposed that the neurotransmitter norepinephrine, released by the sympathetic nervous system, may enhance the expression of SGLT-2. This, in turn, leads to an increase in the reabsorption of glucose and sodium in the renal tubules, as well as elevated levels of blood sugar and blood pressure (73). It has also been demonstrated that dapagliflozin can reduce the expression of tyrosine hydroxylase and norepinephrine in hypertensive mice. These findings suggest that the effect of SGLT-2 inhibitors in protecting renal function could be attributed to a decrease in renal sympathetic nerve activity.

### 3.5 Improve vascular endothelial function

DKD is a complication of diabetes mellitus characterized by damage to the small blood vessels. The dysfunction of the vascular endothelium is considered the initial factor contributing to the development of diabetic microangiopathy. Impaired function of endothelial cell often results in reduced production of nitric oxide (NO) (74, 75). However, extended administration of SGLT-2 inhibitors has been shown to effectively improve vascular endothelial dysfunction in diabetic rats by enhancing NO diastolic function, reducing oxidative stress, and alleviating glucose toxicity in the aortic rings (76, 77). In a study investigating endothelial dysfunction in diabetes, dapagliflozin was found to potentially facilitate the repair of vascular endothelial by decreasing the expression of vascular adhesion molecules, phosphorylated IκB expression, and infiltration of inflammatory macrophages *in vivo* (41).

### 3.6 Reduce uric acid

Chronic hyperuricemia has been consistently identified as a risk factor for the progression of CKD, particularly in individuals with coexisting type 2 diabetes (78). The kidneys play a crucial role in eliminating uric acid from the body, primarily through the renal tubules (79). Within the renal tubules, specific proteins, namely glucose transporter protein 9 (GLUT9) and ATP-binding cassette subfamily G member 2 (ABCG2), are responsible for the reabsorption and excretion of urate, respectively. Of these proteins, GLUT9 plays a central role in the handling of urate (78). The hypouric acid effect of SGLT-2 inhibitors has been linked to the excretion of sugar in the urine (80). These inhibitors work by blocking SGLT receptors, leading to increased glucose excretion in the urine. The excess urinary glucose then competes with GLUT9, reducing the reabsorption of urate and increasing the excretion of uric acid (78). Research has demonstrated that the ability of empagliflozin to lower uric acid levels is associated with the upregulation of ABCG2 expression, which is mediated by the AMPK/Akt/CREB signaling pathway (81).

## 4 Efficacy and safety of SGLT-2 inhibitors in stage G4 DKD

In recent years, the effectiveness and safety of SGLT-2 inhibitors in treating patients with type 2 diabetes and mild to moderate renal insufficiency (eGFR ≥ 30 mL/min/1.73 m^2^) have been confirmed through large clinical trials such as EMPA-REG OUTCOMES (82), DECLARE-TIME 58 (83), CANVAS-R (84), and CREDENCE (85). However, the efficacy and safety of SGLT-2 inhibitors in patients with stage G4 and even G5 DKD have remained unclear until the release of the results from DAPA-CKD (9) and EMPA-KIDNEY (10). These studies have provided a significant basis for establishing the effectiveness and safety of SGLT-2 inhibitors in patients with stage G4 DKD.

### 4.1 Effect on cardiac and renal outcomes

The DAPA-CKD clinical trial enrolled patients with CKD and an eGFR ranging from 25 to 75 mL/min/1.73 m^2^, with up to 67.5% of them having DKD. During the study, which had a median follow-up duration of 2.4 years, the results demonstrated a significant reduction in the risk of the primary endpoint event, which included sustained reduction in eGFR exceeding 50%, ESRD, and a composite endpoint of death resulting from renal or cardiovascular causes in the dapagliflozin treatment group (9). In the subgroup analysis of patients with stage 4 CKD, the use of dapagliflozin group demonstrated significant benefits. The dapagliflozin group exhibited a 27% reduction in the risk of experiencing the main composite endpoint event, which included sustained decline in eGFR exceeding 50%, ESRD, and death from kidney disease. Additionally, there was a 29% decrease in the risk of experiencing sustained decline in eGFR > 50%, ESRD, and death from kidney disease.The risk of hospitalization for heart failure or cardiovascular death was lowered by 17%, and the risk of all-cause mortality decreased by 32%. Furthermore, dapagliflozin treatment also resulted in a slower rate of decline in eGFR and significantly reduced proteinuria. Remarkably, there were no significant differences in therapeutic benefits and safety observed between stage 2-3 CKD and stage 4 CKD groups (86).

The EMPA-KIDNEY trial, with a median follow-up of 2 years, included 46% of patients with DKD and 34.5% of patients with stage 4 CKD and an eGFR of 20–30 mL/min/1.73 m^2^. The primary endpoint events were cardiovascular death or progression of kidney disease. Progression of kidney disease was defined as a > 40% reduction in eGFR from baseline values, a sustained decrease in eGFR to < 10 mL/min/1.73 m^2^, ESRD, or death due to kidney disease causes. The results of the trial showed that the group receiving empagliflozin had a 28% lower risk of cardiac and kidney endpoints compared to the placebo group. Furthermore, there was a 14% lower risk of hospitalization for any reason in the empagliflozin group. Additionally, empagliflozin treatment delayed the decline in eGFR and reduced albuminuria, particularly in patients with a higher urine protein to creatinine ratio at baseline.These beneficial outcomes were consistent across subgroups defined by different eGFR ranges (10). Overall, the EMPA-KIDNEY trial demonstrates that empagliflozin is effective in reducing the risk of cardiovascular and kidney events, hospitalization, and progression of kidney disease in patients with DKD and CKD stage 4. These findings highlight the potential benefits of empagliflozin in managing kidney disease in these patient populations.

In the CREDENCE trial, which focused on patients with DKD, the primary endpoint was defined as doubling of blood creatinine levels, development of ESRD, or death from cardiovascular or renal causes. After 2.6 years of follow-up, the results indicated that canagliflozin reduced the risk of the primary outcome by 30% and the risk of progressing to ESRD by 32% (85). Interestingly, in the subgroup of patients with stage G4 DKD, canagliflozin did not significantly improve glycated hemoglobin levels. However, it did lead to a significant 33% reduction in urinary albumin levels and a slower decline in eGFR compared to the placebo group. These findings were consistent with the DAPA-CKD and EMPA-KIDNEY trials, where the risk of major outcomes in the stage G4 DKD subgroup was similar to that of other subgroups defined by eGFR (87). Overall, these results highlight the efficacy of canagliflozin in reducing the risk of renal and cardiovascular events, as well as slowing the progression of kidney disease in patients with DKD, including those with stage G4 DKD.

The SCORED trial aimed to evaluate the effects of sotagliflozin in patients with DKD, specifically those with an eGFR of 25-65 mL/min/1.73 m^2^. The trial had a median follow-up period of 16 months. The primary endpoint was the incidence of serious cardiovascular adverse events. Additionally, the advancement of renal disease was assessed as a secondary endpoint, which included a decline in eGFR of more than 50% from baseline, ESRD, dialysis, or renal transplantation. However, the results of the trial did not demonstrate statistically significant differences in either all-cause mortality or the renal composite endpoint outcomes between the sotagliflozin group and the control group (88). Surprisingly, the subgroup analysis focusing on patients with stage G4 DKD did not show substantial reductions in the primary endpoints of cardiovascular death and heart failure (89).

A separate study investigating the effects of sotagliflozin over a 52-week period showed that it did not significantly improve glycated hemoglobin levels after 26 weeks. Furthermore, its cardiorenal outcomes were consistent with the findings from the SCORED trial (90). Notably, sotagliflozin did not demonstrate a significant reduction in the risk of cardio-renal outcomes specifically in patients with stage G4 DKD, which is in contrast to the positive results observed in other trials such as DAPA-CKD, EMPA-KIDNEY, and CREDENCE studies. To summarize, SGLT-2 inhibitors have been found to offer significant cardiac and renal benefits to patients with stage G4 DKD, which were comparable to those observed in patients with stage G1-G3 DKD. It is widely acknowledged that as the eGFR decreases, patients experience a decline in glomerular filtration function and a reduced ability to reabsorb glucose and Na^+^ in the renal tubules.

Therefore, their ability to lower blood sugar, improve glomerular hyperfiltration, and reduce volume load is diminished. However, even in the presence of a significant decrease in eGFR, SGLT-2 inhibitors still provide notable cardiorenal benefits. These benefits may be attributed to potential renoprotective effects, such as reducing inflammation and fibrosis, improving oxidative stress, enhancing mitochondrial energy supply, inhibiting the SNS, promoting EPO production, and improving vascular endothelial function. Additionally, considering that sotagliflozin is less effective in improving cardiovascular and renal outcomes in patients with more advanced DKD, dapagliflozin, empagliflozin, and canagliflozin are recommended for the treatment of patients with relatively advanced DKD (**Table 1**).

**Table 1:** Effect of SGLT-2 inhibitors on cardiac and renal outcomes.

Drug	DAPA-CKD	EMPA-KIDNEY	CREDENCE	SCORED
	Dapagliflozin	Empagliflozin	Canagliflozin	Sotagliflozin
Median follow-up(years)	2.4	2.0	2.6	1.3
eGFR(ml/min/1.73m^2^)	25-75	20-90	30-90	25-60
Stage 4 CKD rate	14%(n=624)	34.2%(n=1131)	4%(n=174)	7.7%(n=813)
Renal outcomes Hazard Ratio(95%CI) P Value	0.56(0.45-0.68) p<0.001	0.72(0.64-0.82) P<0.001	0.66(0.53-0.81) P<0.001	0.71(0.46-1.08) p>0.05
Cardiovascular outcomes Hazard Ratio(95%CI) P Value	0.71(0.55-0.92) P=0.009	0.84 (0.64-0.82) P=0.15	0.69(0.57-0.83) P<0.001	0.74(0.63-0.88) P<0.001
Deaths from any cause Hazard Ratio(95%CI) P Value	0.69(0.53-0.88) P=0.004	0.87(0.70-1.08) P=0.21	0.83(0.68-1.02) p>0.05	0.99(0.83-1.18) p>0.05

### 4.2 Adverse reactions

When evaluating the safety of SGLT-2 inhibitors in patients with stage G4 DKD, it is crucial to consider both their positive impact on the heart and kidneys, as well as any potential negative side effects they may cause. Various types of SGLT-2 inhibitors exhibit different adverse events, particularly hypoglycemia, genitourinary infection, ketoacidosis, acute kidney injury (AKI), fractures or amputations, and other adverse events. Therefore, when utilizing SGLT-2 inhibitors, a thorough assessment of the patient’s condition should be conducted.

#### 4.2.1 Hypoglycemia

Since the primary mechanism of SGLT-2 inhibitors involves inhibiting renal glucose excretion, which is independent of insulin secretion, the risk of hypoglycemia is low (91). Clinical trials such as DAPA-CKD, EMPA-KIDNEY, SOCRED, and CREDENCE have demonstrated no increased risk of hypoglycemia in patients with stage G4 DKD (9, 10, 85, 88). Additionally, a study involving type 2 diabetic patients with CKD stages 3b-4 also found no severe hypoglycemic events in the dapagliflozin group, while three (4.3%) severe hypoglycemic events occurred in the placebo group (21). Therefore, SGLT-2 inhibitors have not been shown to raise the incidence of hypoglycemia in patients with advanced DKD.

#### 4.2.2 Ketoacidosis

Ketoacidosis, which is often characterized by gastrointestinal symptoms such as nausea, vomiting, and diarrhea, is a serious adverse effect of SGLT-2 inhibitors (92). The occurrence of ketoacidosis may have a multifaceted mechanism. On one hand, the reduction in blood glucose induced by SGLT-2 inhibitor inhibits insulin secretion. On the other hand, the mechanisms associated with SGLT-2 inhibitors also lead to an increase in glucagon secretion, resulting in a high glucagon/insulin ratio. This elevated ratio plays a significant role in promoting hepatic fatty acid oxidation and ketone bodies production (93). Additionally, the loss of glucose in the kidneys promotes the secondary reabsorption of ketone bodies, further contributing to ketoacidosis (94).

In a recent multicenter study, the risk ratios for ketoacidosis were found to be 3.58, 2.52, and 1.86 for canagliflozin, empagliflozin, and dapagliflozin respectively. The high incidence of ketoacidosis with canagliflozin is primarily attributed to its greater selectivity for SGLT-1 over SGLT-2. Inhibition of SGLT-1 contributes to the development of diarrhea and volume deficit, which can serve as important trigger for ketoacidosis (95). In both the EMPA-KIDNEY and SOCRED trials, ketoacidosis occurred more frequently in the empagliflozin and sotagliflozin groups. Adverse events of diarrhea were also more common in the sotagliflozin group, further increasing the risk of ketoacidosis (10, 88). Therefore, individuals taking SGLT-2 inhibitors should be vigilant for potential ketoacidosis if they experience gastrointestinal symptoms.

#### 4.2.3 Genitourinary system infections

SGLT-2 inhibitors, by promoting the excretion of high amounts of sugar in urine through the kidneys, can increase the risk of urinary tract infections, particularly Candida infections. These infections are more common in females than males and can typically be alleviated with conventional drug therapy (96). Findings from the SCORED trial indicate a higher incidence of genital fungal infections in the sotagliflozin group compared to the placebo group, and this difference was statistically significant (88). Furthermore, other related studies have also suggested a higher likelihood of genital fungal infections in patients with type 2 diabetic CKD stages 3b-4 who use dapagliflozin (21).

#### 4.2.4 Acute kidney injury

Previous explanations for SGLT-2 inhibitor-induced AKI have often focused on the diuretic effect of these inhibitors, which can lead to a decrease in eGFR due to constriction of the small incoming glomerular arteries through tubular feedback (97). However, as research progressed, a more plausible mechanism for the development of AKI was proposed. SGLT-2 inhibitors cause uric aciduria by enhancing the excretion of uric acid via GLUT9 in the renal proximal tubules. This, in turn, triggers an immune response through the activation of inflammatory vesicles and localized inflammatory reactions, ultimately resulting in AKI (98). Additionally, when SGLT-2 inhibitors inhibit glucose reabsorption in the S1 and S2 segments of the renal tubules, there is an increased influx of glucose into the S3 segment. Conversely, the uninhibited SGLT1 receptors also facilitate greater glucose uptake, which may activate aldose reductase and lead to the conversion of glucose in the S3 segment into fructose and sorbitol.

The conversion of glucose in the S3 segment into fructose through fructose kinase activity contributes to the local production of uric acid, oxidative stress, and the release of chemokines, which promote AKI (99). Moreover, the accumulation of sorbitol and fructose due to decreased inositol levels in hyperglycemia may also predispose individuals to the development of AKI (100). Although some studies suggest a potential risk of AKI with SGLT-2 inhibitors, a larger body of research, including studies involving patients with advanced diabetic nephropathy, has failed to demonstrate an increased risk of AKI. The DAPA-CKD trial has indicated that the decrease in serious adverse events related to AKI may reflect the potential protective effect of dapagliflozin beyond the initial decline in eGFR (9). Similarly, the application of empagliflozin, sotagliflozin, and canagliflozin in patients with relatively advanced CKD has also shown no elevated risk of AKI (10, 85, 88). Hence, further studies are needed to confirm whether SGLT-2 inhibitors actually increase the incidence of AKI.

#### 4.2.5 Fracture and amputation

According to the findings from the CANVAS clinical study, the group treated with canagliflozin exhibited a significantly higher incidence of fracture and amputation compared to the placebo group, with incidences that were 97% and 26% higher, respectively. However, the exact mechanism by which SGLT-2 inhibitors contribute to fractures and amputations remains unclear. There have been suggestions that hypovolemia caused by osmotic diuresis and altered bone metabolism due to increased phosphate uptake may be the primary factors responsible for fractures (101, 102). Similarly, hypovolemia and reduced blood flow to the lower extremities resulting from diuresis are thought to be the main causes of amputation (103). In contrast, no increased risk of fracture or amputation was observed in the CREDENCE trial involving canagliflozin (85). Likewise, the DAPA-CKD, EMPA-KIDNEY, and SOCRED trials also found no elevated risk of fracture or amputation (9, 10, 88). After evaluating the potential risks of amputation linked to the use of SGLT-2 inhibitors, the panel reached the conclusion that canagliflozin was the only specific medication associated with an increased risk. They found no evidence suggesting that other SGLT-2 inhibitors posed a similar risk of amputation (104).

Following a comprehensive assessment of the extended effectivenes and adverse events of four SGLT-2 inhibitors in patients with stage G4 DKD, over a follow-up period ranging from 1.3 to 2.6 years, it has been observed that these medications exhibit reduced efficacy in lowering blood glucose levels in such individuals. Additionally, they are associated with adverse events, including urinary tract infections, as well as potential serious risks such as ketoacidosis, fractures, and amputations. However, instances of hypoglycemia and AKI are infrequent. Moreover, most SGLT-2 inhibitors significantly decrease the likelihood of cardiovascular and renal complications in both diabetic and non-diabetic patients with kidney disease over long-term treatment. Consequently, SGLT-2 inhibitors represent a valuable drug option for managing stage G4 DKD in the long run. Furthermore, findings from a 4-year study focusing on patients with diabetes indicate that dapagliflozin demonstrates favorable safety and tolerability profile when used over an extended duration (105). In general, the long-term safety and tolerability of SGLT-2 inhibitors in patients with G4 DKD were found to be relatively favorable. However, it is important to note that the four primary clinical study populations may not fully represent patients with DKD, particularly those in stages G4-5. Therefore, further studies are required to evaluated the effectiveness and safety of SGLT-2 inhibitors specifically in this patient population.

### 4.3 Advantages and disadvantages of individual SGLT-2 inhibitors

#### 4.3.1 Dapagliflozin

Dapagliflozin, the first SGLT-2 inhibitor approved for the treatment of type 2 diabetes (106), continues to demonstrate significant benefits in reducing renal and cardiovascular outcomes, as well as the risk of mortality in patients with stage G4 DKD. Furthermore, its long-term use does not appear to increase the incidence of adverse events, even when patients have an eGFR as low as 15 mL/min/1.73 m^2^. Regarding adverse events, the incidence of serious adverse events was found to be 34.5%. However, the dapagliflozin group did not show an increased risk of adverse events such as hypoglycemia, ketoacidosis, AKI, fracture, and amputation compared to the DKD dapagliflozin group in stages G2-3 or the placebo group in stage G4 DKD. It is worth noting, however, that the incidence of renal-related adverse events was higher in patients with stage G4 DKD.

#### 4.3.2 Empagliflozin

In the EMPA-KIDNEY trial, it was observed that empagliflozin had a beneficial effect in reducing the risk of renal outcomes. However, it did not show a significant reduction in the risk of cardiovascular outcomes or death. Regarding adverse events, the incidence of serious adverse events was found to be 35.2%. It is worth noting that while there is a potential risk of genitourinary infection and ketoacidosis, there was no increased risk of fracture or amputation.

#### 4.3.3 Canagliflozin

In the CREDENCE trial, canagliflozin demonstrated a reduction in the risk of both renal outcomes and cardiovascular outcomes. However, it did not show a significant reduction in the risk of all-cause mortality. With regard to adverse events, the incidence of serious adverse events was found to be 33.5%. The most common serious adverse events were urinary tract infections (13.4%) and ketoacidosis (0.5%). Importantly, there was no increased risk of fracture or amputation associated with canagliflozin usage.

#### 4.3.4 Sotagliflozin

In the SOCRED trial, sotagliflozin showed a reduction in cardiovascular outcomes risk. However, it did not demonstrate a reduction in renal or cardiovascular risk specifically in the stage G4 DKD subgroup. In terms of adverse events, the incidence of serious adverse events was 23.4%. Diarrhea (8.5%), ketoacidosis (0.6%), and urinary tract infections (11.5%) were more frequently reported with sotagliflozin compared to placebo. Importantly, there was no increased risk of fracture or amputation observed.

In summary, all four SGLT-2 inhibitors have shown the ability to reduce renal, cardiovascular, and mortality risks in patients with stage G4 DKD, with generally favorable safety profiles. Among them, dapagliflozin stands out as it effectively reduces renal, cardiovascular, and mortality risks without the occurrence of ketoacidosis, and it also lowers the incidence of AKI. However, sotagliflozin appears to be less effective in improving cardiac and renal outcomes in advanced DKD patients and should be used with caution in this population. In terms of adverse events, sotagliflozin has the lowest incidence of serious adverse events, but is more associated with diarrhea and urinary tract infections. It is worth noting that, except for dapagliflozin, the other three SGLT-2 inhibitors carry a potential risk of ketoacidosis. Therefore, dapagliflozin is recommended as a preferable option for patients with advanced DKD.

## 5 Conclusion and Prospect

Delaying the progression of diabetic nephropathy, a leading cause of ESRD, is of utmost importance. SGLT-2 inhibitors, a novel class of glucose-lowering drugs, have shown substantial cardiovascular and renal protective effects in patients with stage 1-4 CKD, regardless of the presence of diabetes. However, as eGFR declines, the hypoglycemic effects, improvement of glomerular hyperfiltration, and suppression of the RAAS by SGLT-2 inhibitors become less effective. Despite this, SGLT-2 inhibitors continue to demonstrate significant cardiorenal benefits in advanced DKD, potentially due to their ability to suppress inflammation and fibrosis, improve oxidative stress, enhance EPO production, optimize mitochondrial energy supply, inhibit the SNS, and protect vascular endothelial cells. These mechanisms likely contribute to the observed renal protective effects of SGLT-2 inhibitors in advanced DKD.

Further investigation and clarification are needed to fully understand the linked mechanisms and the renal protective effects of SGLT-2 inhibitors from multiple perspectives. Currently, the efficacy and safety of SGLT-2 inhibitors in treating patients with advanced DKD have been increasingly supported. The threshold for using SGLT-2 inhibitor has been lowered to an eGFR of 20 mL/min/1.73 m^2^, and they can be continued until the initiation of dialysis or renal transplantation, as long as they are well-tolerated by the patient. It is exciting to anticipate whether future advancements will relax the restrictions on the use of SGLT-2 inhibitors for patients with all stages of CKD.

## Author contributions

J-XT and C-WY formulated and conceived of this study. Z-CD, J-XC, RZ, X-BL, J-XT, and C-WY wrote the manuscript. All authors contributed to the article and approved the submitted version.
